# Enhanced Delivery of Neuroactive Drugs via Nasal Delivery with a Self‐Healing Supramolecular Gel

**DOI:** 10.1002/advs.202101058

**Published:** 2021-05-24

**Authors:** Julie Tzu‐Wen Wang, Ana C. Rodrigo, Anna K. Patterson, Kirsten Hawkins, Mazen M. S. Aly, Jia Sun, Khuloud T. Al Jamal, David K. Smith

**Affiliations:** ^1^ Institute of Pharmaceutical Science, School of Cancer and Pharmaceutical Sciences, Faculty of Life Science and Medicine King's College London 150 Stamford street London SE1 9NH UK; ^2^ Department of Chemistry University of York Heslington York YO10 5DD UK

**Keywords:** drug delivery, hydrogel, neuroactive drugs, supramolecular gel

## Abstract

This paper reports the use of a self‐assembling hydrogel as a delivery vehicle for the Parkinson's disease drug l‐DOPA. Based on a two‐component combination of an l‐glutamine amide derivative and benzaldehyde, this gel has very soft rheological properties and self‐healing characteristics. It is demonstrated that the gel can be formulated to encapsulate l‐DOPA. These drug‐loaded gels are characterized, and rapid release of the drug is obtained from the gel network. This drug‐loaded hydrogel has appropriate rheological characteristics to be amenable for injection. This system is therefore tested as a vehicle for nasal delivery of neurologically‐active drugs—a drug delivery strategy that can potentially avoid first pass liver metabolism and bypass the blood–brain barrier, hence enhancing brain uptake. In vitro tests indicate that the gel has biocompatibility with respect to nasal epithelial cells. Furthermore, animal studies demonstrate that the nasal delivery of a gel loaded with ^3^H‐labeled l‐DOPA out‐performed a simple intranasal l‐DOPA solution. This is attributed to longer residence times of the gel in the nasal cavity resulting in increased blood and brain concentrations. It is demonstrated that the likely routes of brain penetration of intranasally‐delivered l‐DOPA gel involve the trigeminal and olfactory nerves connecting to other brain regions.

## Graphical abstract

A self‐assembling gel based on a low‐molecular‐weight gelator is demonstrated to achieve effective intranasal delivery of the Parkinson's disease drug l‐DOPA. In particular, using the supramolecular gel enhances residence times in the nasal cavity and improves delivery of the drug both into the blood and brain compared with a simple solution of l‐DOPA administered the same way.

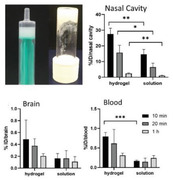

## 1 Introduction

Supramolecular gels are tunable and responsive soft materials in which a “solid‐like” network self‐assembled from a low‐molecular‐weight gelator (LMWG) immobilises a “liquid‐like” phase.^[^ ^1^ ^]^ They have wide‐ranging potential high‐tech applications from environmental remediation to regenerative medicine.^[^ ^2^ ^]^ Gels based on LMWGs have particular potential in the field of drug delivery as a result of their ability to encapsulate active pharmaceutical ingredients (APIs) within their structures and control their release in a variety of ways.^[^ ^3^ ^]^ If the API is a part of, interacts with, or is physically trapped within the self‐assembled “solid‐like” LMWG network, then its release can be relatively slow, with gel erosion or triggered disassembly being required to break down the gel and release the API.^[^ ^4^ ^]^ It is, however, also possible that the API is only physically encapsulated within the gel, and is thus freely dissolved in the mobile “liquid‐like” phase—in this case, API release can be more rapid, with the gel acting primarily as a formulation tool.^[^ ^5^ ^]^ In terms of patient administration, gels are most often considered for delivery by injection—for example subcutaneously or intratumorally, with the goal of controlled slow release.^[^ ^6^ ^]^ Topical and transdermal delivery by application of self‐assembled gels onto the skin is also of considerable interest,^[^ ^7^ ^]^ often with the goal of achieving effective delivery via non‐invasive means and avoiding first pass liver metabolism.

In recent years, nasal delivery has become an increasingly interesting drug administration mode.^[^ ^8^ ^]^ Nasal delivery has the potential to achieve rapid systemic uptake via absorption into the bloodstream through nasal epithelia.^[^ ^9^ ^]^ Furthermore, the accessibility of the olfactory and trigeminal nerves opens the possibility of direct brain delivery without the need to cross the blood–brain barrier.^[^ ^10^ ^]^ With the rise in incidence of neurological diseases associated with ageing, such as Parkinson's and Alzheimer's diseases, the ability to achieve direct delivery of pharmaceutically‐active agents into the brain is of considerable importance.^[^ ^11^ ^]^ Taking Parkinson's disease as an example, there are significant difficulties with treatment.^[^ ^12^ ^]^ The standard medication, based on oral l‐DOPA‐carbidopa administration, is subjected to first pass metabolism prior to uptake into the central nervous system, resulting in very small amounts of the drug reaching the brain. Furthermore, drug metabolism is up‐regulated on prolonged exposure to the drug, meaning that patients with this chronic condition must progress to increasingly higher doses of the drug, and ultimately receive the “rescue” pump‐based direct delivery of l‐DOPA‐carbidopa intestinal gel into the jejunum in an attempt to overcome some of the metabolic problems.^[^ ^13^ ^]^ Direct brain delivery of an active drug clearly has the potential to be transformative for such patients.

In terms of achieving the best outcomes for nasal drug delivery, enhancing residence times in the nasal cavity, and improving contact between the API and nasal epithelia or the olfactory nerve are important and common strategies in enhancing the degree of uptake, with modification of the dosage form (e.g., emulsion formulations) or incorporation with mucoadhesive agents (e.g., pectin and chitosan polymers) in the formulation.^[^ ^14^ ^]^ We reasoned that supramolecular gels could be ideally suited to this application, given their potential for self‐healing and self‐assembly in situ, combined with their soft rheological properties. Although there has been rising interest in the use of responsive polymer gels for nasal drug delivery,^[^ ^15^ ^]^ perhaps surprisingly, in spite of the attention focused on low‐molecular‐weight gelators,^[^ ^3^ ^]^ they have not previously been explored in this regard. There have been isolated reports in which l‐DOPA has been incorporated in different ways into supramolecular gels,^[^ ^16^ ^]^ but these have not been applied in a pharmaceutical setting.

With nasal delivery in mind, we reasoned that the ideal supramolecular hydrogel would need shear thinning and recovery capacity, so that it can be administered to the nose via injection or spraying as a solution, and then form a thin gel film in situ, coating nasal epithelia. Furthermore, the gel should be based on simple components with low toxicity, and also have the potential to easily degrade into non‐self‐assembling units, limiting the risk of stable self‐assembled structures building up in vivo on long‐term exposure to the delivery vehicle. Finally, the gel should be compatible with neurologically‐active drugs (we chose in this study to focus on l‐DOPA as a model drug) and be capable of rapid drug release. The goal is not to achieve slow release of the drug in the nasal cavity, or a permanent thin layer of gel on the nasal epithelia. Indeed this would be both challenging and undesirable as mucus is rapidly cleared from the nasal cavity, meaning that in nasal drug delivery, a clearance half life of about 15 min is typical.^[^ ^17^ ^]^ As such, rapid release of the API from the gel is important so that drug release kinetics do not become the limiting factor in the overall delivery capacity of the system. In comparison to previously studied polymer gel systems, supramolecular gels offer advantages of being fully reversible materials that are easily modified synthetically, whilst retaining the potential to be formulated with a wide range of additives that might potentiate drug delivery and stability. This paper reports the results of our initial studies, the development of a first generation LMWG for this application, and through in vivo studies, demonstrates the potential of supramolecular gels to enhance nasal residence times and potentiate the delivery of APIs via this attractive mode of administration.

## 2 Results and Discussion

**Figure 1:**
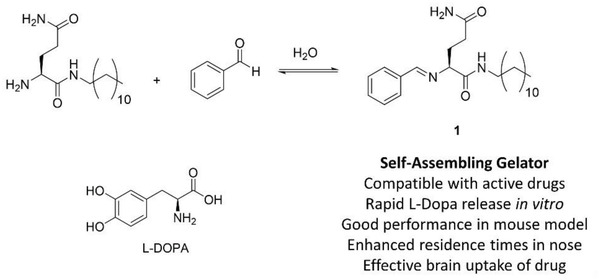
Synthesis of gelator **1** by mixing a glutamine amide derivative and benzaldehyde in water, structure of l‐DOPA and advantages of the self‐assembling gelator approach for nasal delivery.

Based on the design criteria above, we selected our recently disclosed two‐component gelation system (**1**) as a potentially suitable LMWG .^[^ ^18^ ^]^ This system assembles into gels when a glutamine amide derivative and benzaldehyde are mixed together in a 1:1 ratio, with a reversible reaction between the two‐components giving rise to Schiff base **1**, which is responsible for self‐assembly and gelation (**Figure** **1**). We reasoned this gelator was an ideal candidate for drug delivery applications as the Schiff base should be non‐persistent over long timescales in vivo as a result of hydrolysis, potentially limiting the deposition of any self‐assembled material on repeated administration. This gelator will break down into the glutamine amide derivative and benzaldehyde. Benzaldehyde is generally regarded as safe,^[^ ^19^ ^]^ being used at concentrations up to 0.5% in some perfumes and also as a flavoring.^[^ ^20^ ^]^ Nasal irritation has been observed under extreme conditions, on extended inhalation of volatilized benzaldehyde at 500 ppm for repeated 6 h periods.^[^ ^21^ ^]^ However, it is rapidly metabolized to benzoic acid and has very little acute toxicity.

We initially determined the amount of free benzaldehyde in our two‐component gel. Our standard gel was made by mixing 3.5 mg of glutamine amide derivative and 1.15 mg (1.1 µL) of benzaldehyde in 1 mL of water. This constitutes a benzaldehyde loading of 0.115% w/v, well below the concentration at which benzaldehyde is used in (e.g.) perfumes. We then employed ^1^H NMR on the self‐assembled gel in order to detect the mobile components present within the liquid‐like phase—by using ^1^H NMR on gels, the self‐assembled components of the “solid‐like” nanofibers are not detected, and only the solution‐phase mobile components are visualized (e.g., Figure S1a, Supporting Information).^[^ ^22^ ^]^ The use of a known mass of an internal standard (dimethylsulfoxide, DMSO), which is also mobile in the liquid‐like phase, therefore allows the quantification of any non‐assembled material. In this way, we were able to determine that after gel assembly, ≈35% of the benzaldehyde was observed as sharp NMR peaks, and hence could be assigned as being not incorporated in the gel nanofibers. This therefore constitutes a concentration of “free benzaldehyde” within the gel of 0.04% wt vol^−1^ (400 ppm). ^1^H NMR studies over time indicated that the free benzaldehyde fell to ≈9% (0.01%, 100 ppm). It is also important to note that this “free” benzaldehyde is in viscous aqueous solution and therefore is not strictly volatilized within the nasal cavity and will not be problematic for rapid nasal delivery. We have also previously shown that gels can still effectively be formed using only 0.7 equiv. of benzaldehyde,^[^ ^18^ ^]^ and using this approach, effectively all of the benzaldehyde is immobilized within the solid‐like gel nanofibers. We were therefore comfortable that this two‐component gel constituted a reasonable platform for further investigation as a nasal delivery system and that the impact of benzaldehyde could be minimized.

**Figure 2:**
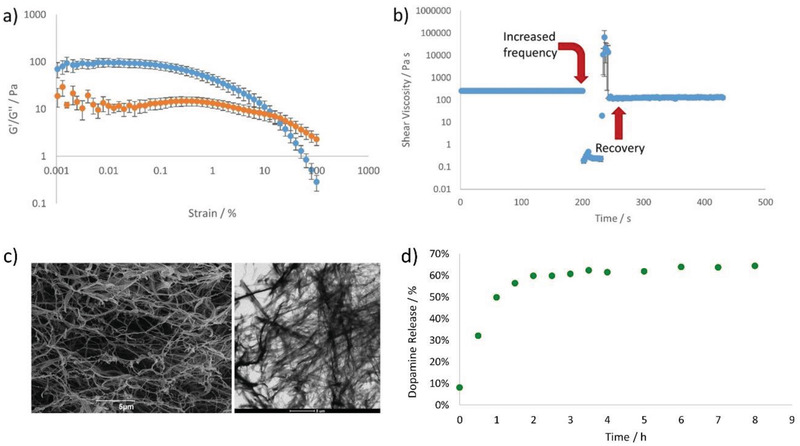
a,b) Rheological study of gel formed by compound **1** (0.46% w/v) in water showing a) response of storage and loss modulus (G′, blue and G″, orange) to strain and b) the shear viscosity over time. At 200 s, a large strain is applied to the sample, which was then left to recover its gel‐like characteristics—this process took 40 s. c) (Left) SEM image of hydrogel formed from **1** loaded 1:1 with l‐DOPA, (right) TEM image of hydrogel formed from **1** loaded 1:1 with l‐DOPA. d) Release of l‐DOPA in vitro from the hydrogel formed by **1** loaded with l‐DOPA (0.8 x 10^–3^ m).

This hydrogel was characterized in some detail in our previous report,^[^ ^18^ ^]^ but it was important here to demonstrate that the gel had suitable rheological properties to make it a candidate for nasal delivery. Rheology was performed using a parallel plate geometry and as expected for a gel, *G*′ > *G*′′, demonstrating that the solid‐like characteristics dominate (**Figure** **2**a). The frequency sweep indicated that this gel was very soft indeed, with a *G*′ value of just 85 Pa. Furthermore, the gel broke down at ≈12.5% strain, and had a short linear viscoelastic region with the tendency to behave like a viscous liquid. We reasoned that these properties were suitable for potential nasal delivery, where a thin coating of viscous material on the walls of the nasal cavity is required. It has also previously been shown that softer gels can enhance skin delivery compared with more rigid systems.^[^ ^23^ ^]^

Most importantly, for the delivery of a gel into the nasal cavity, the gel must rapidly assemble in situ after spraying or injecting in liquid form. We noted experimentally, that on injection through a syringe, this gel initially exhibited shear thinning behavior and acted as a solution, however, on standing after injection, a full sample‐spanning gel would once again form. This type of “self‐healing” capacity^[^ ^24^ ^]^ would be a key requirement of a gel for nasal delivery, as it will allow the gel to be applied to the nasal cavity, for example via a nasal spray, and then reform a gel in situ to ensure effective contact with the nasal epithelium. We therefore determined the shear thinning and recovery of this gel in more detail. In particular, we were interested in the kinetics of this process to ensure it had the potential to be pharmaceutically useful. Characterization was achieved using rheological methods in which a gel was formed on the plate and a shear force of 0.0126% applied at a frequency of 2 Hz. After 200 s, the frequency was increased to 100 Hz for 30 s, leading to shear thinning and visible destruction of the gel. The frequency was then lowered back to 2 Hz, and the recovery of the gel was monitored over time (Figure 2b, Figure S2, Supporting Information). Pleasingly, the gel recovered much of its rheological performance within ≈10 s as indicated by the recovery of *G*′, the reformation of a soft gel‐like material in which *G*′ > *G*′′ and the visible re‐assembly of a gel on the rheometer. We chose this methodology because spraying is typically performed by application of high shear frequency,^[^ ^25^ ^]^ and are therefore confident that this gel has appropriate rheological properties for the desired application.

We then went on to determine the ability of the gel to incorporate a model neurologically‐active API. In this study, we chose l‐DOPA, which has the clinical need for improved Parkinson's treatment as described in the Introduction. In our standard formulation, we loaded 3.5 mg of l‐DOPA into the gel (1 mL of water, 3.5 mg of glutamine amide derivative and 1.15 mg of benzaldehyde). We could incorporate l‐DOPA into the gel at loadings up to a 4:1 molar ratio with respect to the benzaldehyde and form transparent gels (Table S1, Supporting Information). We could still form gels at API loadings of 10:1, but not all of the l‐DOPA fully dissolved. In our standard formulation, the presence of l‐DOPA had minimal impact on gel performance. Rheological studies indicated little change in the macroscopic behavior, with *G*′ in the linear viscoelastic region being ≈100 Pa, and the *G*′/*G*′′ crossover point being observed at 7% strain (Figures S3 and S4, Supporting Information). The l‐DOPA‐loaded gel still exhibited thixotropic properties with rapid recovery to the gel phase after application of high frequency shear. Scanning electron microscopy (SEM) and transmission electron microscopy (TEM) imaging indicated minimal perturbation of the self‐assembled nanofibrillar network (Figure 2c and Figure S5, Supporting Information). However, TEM imaging at higher l‐DOPA loadings indicated that aggregates associated with l‐DOPA could also be observed using this technique, consistent with the non‐transparent nature of these gels (Figure S6, Supporting Information).

^1^H NMR spectroscopy on samples of the gels loaded with l‐DOPA was used to provide some insight into the way l‐DOPA is incorporated into the gel. There are several possibilities for active agents that have been formulated into supramolecular gels and these can be distinguished via standard solution‐phase ^1^H NMR methods applied to a sample of gel:

i) The active agent can interact with the “solid‐like” gel nanofibers, or be entrapped within the gel network as a result of its size. In either case, the molecular‐scale mobility of the active agent is restricted, meaning it cannot be observed by ^1^H NMR spectroscopy.
ii) The active agent can be dissolved within the “liquid‐like” phase of the gel, freely able to diffuse through the gel network. In this case, the active agent can be detected by ^1^H NMR in the same way as any dissolved solute.
iii) The active agent can react with the self‐assembled fibers and hence become immobilized in the gel, or lead to disassembly of the material—this was considered possible in this case, as l‐DOPA contains an amine group which could plausibly react with benzaldehyde, although it was considered unlikely under the loading pH conditions.

Adding an internal standard to the gel allows us to quantify the amount of “mobile” active agent, and hence gain effective insight into the molecular‐scale organization of the gel.^[^ ^22^ ^]^ We found that 92% of the l‐DOPA was visible in unmodified form via ^1^H NMR spectroscopy as sharp peaks, quantified using a DMSO internal standard, hence indicating that the active agent is essentially free to diffuse through the gel, and does not significantly interact or react with components of the “solid‐like” self‐assembled network (e.g., Figure S1b, Supporting Information). Although this would be a drawback for applications requiring controlled or slow release (and is something we are currently pursuing in that regard), it is ideal for the proposed nasal delivery mode, as rapid release of the unmodified active ingredient once the gel comes into contact with the nasal epithelium is needed.

We went on to quantify the ability of the gel to release l‐DOPA in vitro. This was achieved by exposing the gel to a supernatant receiving solution at pH 7, and taking aliquots from this supernatant to determine the extent of release via UV–vis spectroscopy. Performance was benchmarked against control gels, in which l‐DOPA was not present. Release of l‐DOPA was rapid (Figure 2d), consistent with the view described above that l‐DOPA does not interact with the “solid‐like” gel nanofibers, but instead is highly mobile within the “liquid‐like” environment of the self‐assembled gel. Not all of the l‐DOPA was released from the gel, and this may indicate that some of the l‐DOPA under these conditions is precipitated in the gel. We anticipate more l‐DOPA would have been released if the supernatant had been replaced with fresh solution, but this is less relevant for rapid nasal delivery. The pattern of rapid release of more than 60% API is suitable for the proposed nasal delivery administration mode. We also tested the stability of the gels in the presence of various relevant additives to aqueous solution (Tables S2 and S3, Supporting Information).

**Figure 3:**
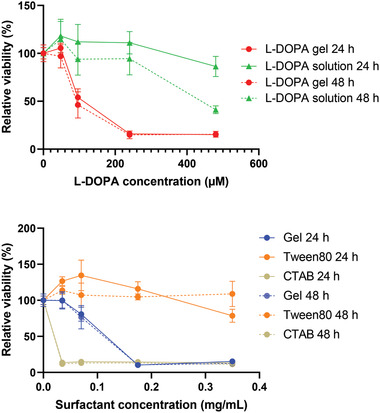
Cytotoxicity of hydrogel and l‐DOPA loaded hydrogel in human nasal septum tumour RPMI 2650 cells. a) Cells were incubated with culture medium containing up to 10% l‐DOPA loading gel and l‐DOPA solution at the equivalent concentrations (up to 480 x 10^–6^ m) for 24 and 48 h. b) Cells were treated with various concentration of hydrogel, Tween 80 and CTAB (up to 0.35 mg mL^−1^) for 24 and 48 h. Data are expressed as mean ± SD, *n* = 5. Cytotoxicity was assessed by the standard MTT assay.

With nasal delivery in mind, we determined experimentally the cytotoxicity of these self‐assembled gels. This was achieved by exposing nasal epithelial cells to the gels in vitro. A gel loaded with 4.8 x 10^–3^ m l‐DOPA was formulated and human nasal septum tumour RPMI 2650 cells were incubated with culture medium containing 1%, 2%, 5%, and 10% gels (v/v) for 24 and 48 h. Cells were treated with l‐DOPA solution prepared in water at the same range of l‐DOPA concentration for comparison. As shown in **Figure** **3**a, some concentration‐dependent cytotoxicity was observed in cells treated with l‐DOPA‐loaded gel for 24 h and 48 h (red solid and dash lines). In contrast, no obvious toxicity was observed in the cells treated with equivalent concentrations of l‐DOPA solution for 24 h and the cell viability only decreased at the highest concentration at 48 h (green solid and dash lines). The results indicated the toxicity was associated mainly with the gel. We suggest this is most likely due to the presence of glutamine amide units disassembled in culture medium and acting as an amphiphile. Complete gel disassembly, however, is an extreme scenario which may not take place, or only occur slowly, upon in vivo nasal administration as the nasal cavity is covered by viscous but not fluid mucus, and the timescales of exposure are very much smaller (minutes rather than hours).

We further investigated the toxicity of the amphiphilic glutamine amide by comparing it against other surfactants such as Tween 80, a known “biologically‐safe” surfactant that has been used widely in food, cosmetics, and drug formulations,^[^ ^26^ ^]^ and cetyltrimethylammonium bromide (CTAB), a highly toxic but common surfactant used as a surface coating agent.^[^ ^27^ ^]^ Cells were treated with plain gel without l‐DOPA at equivalent loadings (up to 0.35 mg mL^−1^). It should be noted that Tween 80 has a much higher molecular weight than the other two surfactants, and a loading of 0.35 mg mL^−1^ is actually equivalent in terms of concentration to a gelator loading of 0.07 mg mL^−1^ (both ≈400 x 10^–6^ m concentration). It was found that the disassembled gel was non‐toxic at low loadings (≈0.07 mg mL^−1^), comparable to Tween 80 at the same concentration, whereas severe toxicity was induced by CTAB at much lower concentrations (Figure 3b). The results therefore indicate some in vitro biocompatibility of the studied gel.

To demonstrate the potential application of gels such as these for intranasal drug delivery, we performed in vivo studies using radiolabeled [^3^H]l‐DOPA and studied the uptake and biodistribution of [^3^H]l‐DOPA hydrogel following intranasal administration in naïve Balb/c mice. Comparative studies were also performed to dose mice with [^3^H]l‐DOPA solution intranasally. Mice were anaesthetized and injected with [^3^H]l‐DOPA hydrogel or solution (l‐DOPA 0.95 mg kg^−1^, 1.5 µCi per mouse). At defined timepoints (10 min, 20 min, 1 h; *n* = 3 for each timepoint), major organs, brain tissue, and nasal cavity were excised following transcardiac perfusion with saline under terminal anesthesia to avoid false positive results from blood. Blood was collected at each time point before perfusion.

**Figure 4:**
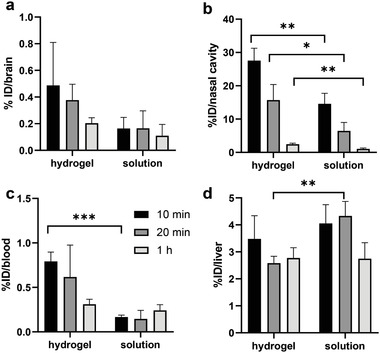
Uptake of [^3^H]l‐DOPA formulated in hydrogel or in solution in a) brain, b) nasal cavity, c) blood, and d) liver up to 1 h post intranasal administration. At the experimental end points, whole body perfusion with 0.9% saline was performed and studied tissues were harvested and proceeded for liquid scintillation counting. Results are expressed as %ID per tissue. Data are expressed as mean ± SD, *n* = 3. Statistical significance is indicated in comparison between each mode at the same time‐point. *p < 0.05, **0.01 < *p* < 0.05; ***p < 0.01 (one‐way ANOVA).

The [^3^H]l‐DOPA levels detected in brain, nasal cavity, and blood following intranasal administration are shown in **Figure** **4**. Overall organ biodistribution profiles of each delivery mode is shown in Figure S7, Supporting Information. In view of brain uptake, intranasal delivery of l‐DOPA with the hydrogel formulation appeared to be more effective (0.49±0.32% ID (injected dose) after 10 min) than that observed in simple solution (0.16±0.08% ID after 10 min), suggesting potential benefits of the gel formulation, presumably due to enhancing residence times in the nose for more effective uptake through nasal epithelia (Figure 4a). Specifically, in support of this hypothesis, 27.55±3.73% ID of l‐DOPA was found in the nasal cavity after 10 min (and 15.70±4.7% ID after 20 min) in the presence of the gel, whereas this falls significantly to just 14.60±3.17% ID after 10 min (and 6.44±2.54% ID after 20 min) when l‐DOPA was delivered as a solution (Figure 4b). The rapid brain adsorption of l‐DOPA when applied intranasally (i.e., within half an hour) has been reported^[^ ^28^ ^]^ and is characteristic for intranasal administration. In addition to the direct nose‐to‐brain route, drugs could also be absorbed indirectly through the blood–brain pathway since the nasal cavity is extensively vascularized. Pharmacokinetic profiles of l‐DOPA in brain run parallel to those in blood after nasal administration in both gel and solution forms (Figure 4a,c). Interestingly, in addition to the significantly enhanced residence in the nasal cavity, there was also a significantly higher level of l‐DOPA in blood at the earliest time point when the gel was present in the intranasal formulation. This is likely also a result of increased retention time in the nasal cavity in the presence of the gel. It is worth noting that the relatively rapid decay of l‐DOPA levels in the brain over the first hour after delivery is mirrored by data found in other studies of intranasal delivery, where, for example the period of peak l‐DOPA concentration was found to be 9–15 min after administration.^[^ ^28a^ ^]^

The intranasal route can ideally bypass first‐pass metabolism in the liver and gastrointestinal (GI) tract, which is the cause of low bioavailability after oral administration, the current standard l‐DOPA treatment. However, GI absorption and metabolism after nasal administration cannot be completely avoided as a portion of drugs might be either cleared into the GI tract by cilia moving towards the nasopharynx or be swallowed.^[^ ^29^ ^]^ We observed that the presence of the gel in the delivery formulation significantly lowered the amount of l‐DOPA found in the liver, particularly at 20 min post administration (Figure 4d). We suggest that when l‐DOPA was injected intranasally as a solution, some of it might be drained to the nasopharynx, ending up in the GI tract and then undergoing the usual route of oral delivery, leading to liver accumulation. We argue that the presence of the gelator has the desired effect of viscosifying the overall formulation in the nasal cavity, hence limiting the flow into the GI tract and preventing drainage to the nasopharynx. This argument is supported by the observation that significantly more l‐DOPA was found within the nasal cavity, and that its persistence there was significantly longer if the gelator is present during intranasal delivery.

We also compared intranasal delivery of l‐DOPA hydrogel with intravenous injection of l‐DOPA solution, which is sometimes prescribed to late‐stage Parkinson's patients who cannot tolerate oral medications. Pleasingly, the results showed that the nasal delivery of gel formulation achieved higher levels in brain (0.49±0.32% ID versus 0.12 ± 0.01% ID) and blood (0.79±0.11% ID versus 0.37±0.24% ID), and significantly lower uptake in the liver compared with intravenous delivery of l‐DOPA at 10 min post administration (Figure S8, Supporting Information). Higher levels of l‐DOPA detected in liver following intravenous injection are expected as a result of considerable metabolism at the peripheral sites^[^ ^30^ ^]^ and our results indicate that this complication can be overcome by intranasal delivery at ease. Nasal delivery with our gel formulation therefore achieves 4.1 times more brain uptake of l‐DOPA and 2.1 times more l‐DOPA in the blood 10 min after administration than intravenous delivery of an equivalent dose. In this study, we did not carry out comparative studies of oral l‐DOPA delivery, however, other published work^[^ ^28a^ ^]^ indicates that IV delivery achieves >10 times the effective concentration in both brain and plasma compared to the oral delivery route. We therefore conclude that our nasal delivery gels would be significantly more effective than oral l‐DOPA delivery.

It is worth reflecting further on the therapeutic relevance of these observations. In a detailed pharmacokinetic study comparing intranasal (2.5 mg kg^−1^ dissolved in maleic acid/hydroxypropyl‐*β*‐cyclodextrin/triethanolamine at pH 2.7) and oral (80 mg kg^−1^, dissolved in saline) delivery of l‐DOPA in rats, it was demonstrated that levels of l‐DOPA in the brain achieved by intranasal delivery were 2.1 to 2.5 times higher than oral delivery even though the dose was 32 times lower.^[^ ^28a^ ^]^ In a therapeutic efficacy study, l‐DOPA applied nasally at a relatively high dose of 12 mg kg^−1^ with the help of oil, improved motor functions and performance in the rotation test using a hemiparkinsonian rat model.^[^ ^28b^ ^]^ In an alternative study using the same hemiparkinsonian rat model, however, a much lower dose of just 0.35 mg kg^−1^ delivered intranasally appeared to be therapeutically effective in the forelimb placing task test 30 min after administration.^[^ ^31^ ^]^ This dosing regime was applied once a day, and the therapeutic effects persisted until treatment was withdrawn. This study therefore suggests the dose given here of 0.95 mg kg^−1^ l‐DOPA to mice (equivalent to 0.475 mg kg^−1^ to rats) does have the potential to be therapeutically effective. Furthermore, there remains scope within our gel delivery system to further increase the dose of l‐DOPA—this preliminary study was primarily aiming to show the benefits of our gel formulation over simple intranasal administration of a solution—the dose and therapeutic effects have not as yet been optimized. In addition, it would be also desirable to optimize our system by including carbidopa (a decarboxylation inhibitor) and/or other delivery vehicles in the gel formulation, which can enhance l‐DOPA half life in the brain and significantly improve therapeutic outcomes^[^ ^28^, ^31^ ^]^—work in this regard is ongoing.

**Figure 5:**
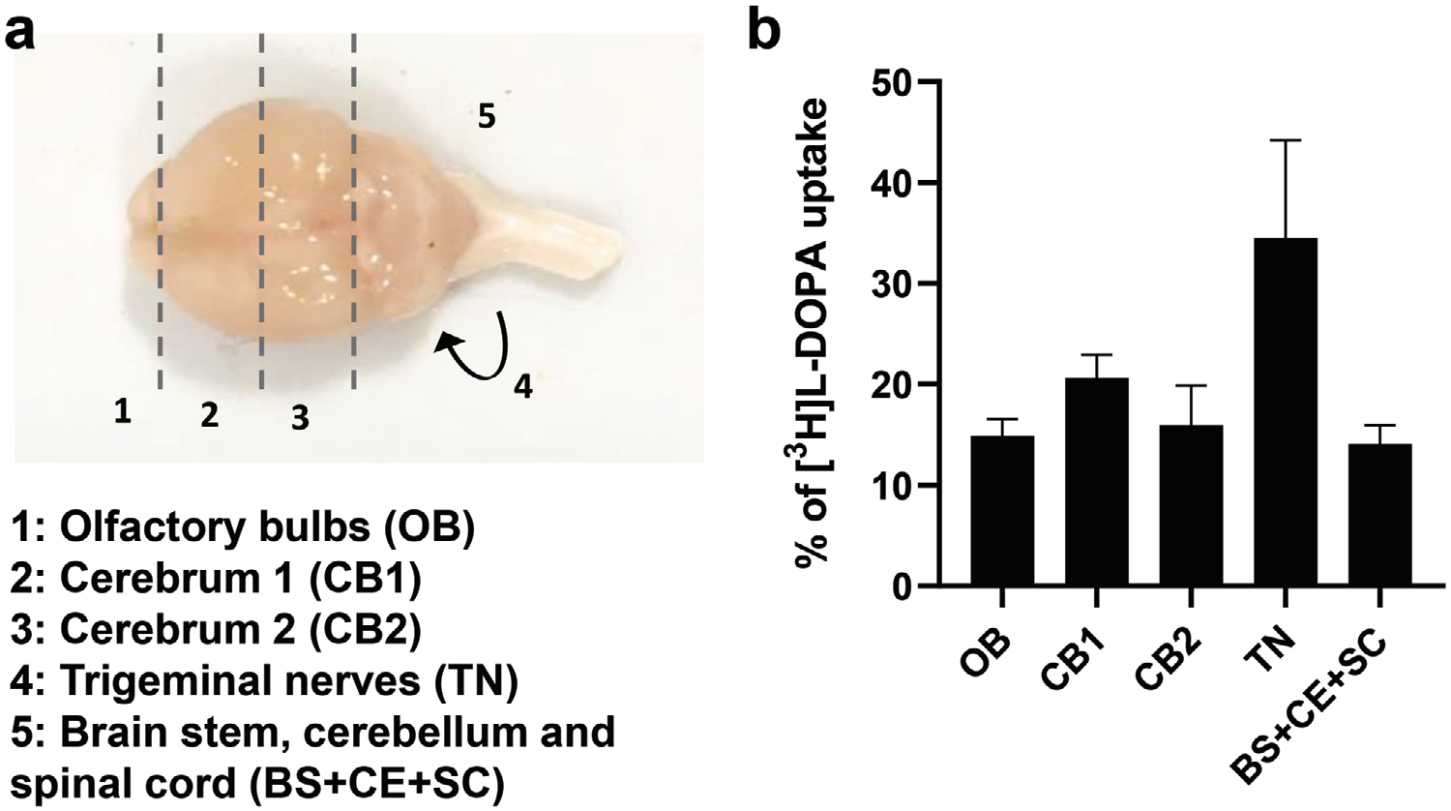
Brain distribution of [^3^H]l‐DOPA hydrogel at 10 min post intranasal administration. a) Dorsal view of the mouse brain and dissection guidance of different brain segments, the olfactory bulbs (OB), the cerebrum (CB 1&2), the brain stem (BS), cerebellum (CE), spinal cord (SP), and trigeminal nerves (TN). b) % of [^3^H]l‐DOPA uptake in different brain segments. At the experimental end points, whole body perfusion with 0.9% saline was performed and studied tissues were dissected and proceeded for liquid scintillation counting. Results are expressed as % uptake normalised to total [^3^H]l‐DOPA detected in these collected tissues. Data are expressed as mean ± SD, *n* = 3.

We next performed a pilot study to investigate the transport pathways of l‐DOPA hydrogel in the brain after nasal administration. We determined the levels of radioactivity in different brain segments, the olfactory bulbs (OB), the cerebrum (CB 1&2), the brain stem (BS), cerebellum (CE), spinal cord (SP) and trigeminal nerves (TN) at 10 min after nasal administration of [^3^H]l‐DOPA hydrogel, the time point with the highest brain uptake observed in the present study, by liquid scintillation analysis (**Figure** **5**a). As shown in Figure 5b, [^3^H]l‐DOPA was distributed over the entire brain and the highest [^3^H]l‐DOPA level was detected in TN among the dissected tissues, containing more than 30% of the total radioactivity detected in the brain. Direct transport of molecules from the nasal cavity to the brain can occur along the olfactory and/or trigeminal nerves, originating in the CB and pons of the BS, respectively.^[^ ^15b^ ^]^ For instance, olfactory transfer for [^3^H]dopamine has been reported and [^125^I]insulin‐like growth factor‐I undergoes both olfactory and trigeminal pathways to reach brain and spinal cord following nasal administration.^[^ ^32^ ^]^ The current data suggest a combination of both pathways accounts for the transport of [^3^H]l‐DOPA hydrogel after nasal delivery and that the trigeminal route may dominate the transport.

In addition to studies referred to above,^[^ ^28^, ^31^ ^]^ early studies have demonstrated that intranasal administration of l‐DOPA methyl ester (higher solubility) solution can increase the extracellular dopamine levels in the ipsilateral neostriatum in rats.^[^ ^33^ ^]^ It has also been proved that intranasally applied l‐DOPA, dispersed in castor oil, can alleviate Parkinson's symptoms using a rat model of Parkinson's disease.^[^ ^34^ ^]^ A recent Phase 2a clinical trial has explored the therapeutic potential for intranasal l‐DOPA for Parkinson's disease by administration of a powder formulation of l‐DOPA using a specific nasal spray device (NCT03541356).^[^ ^35^ ^]^ The results of the present study demonstrate the genuine potential of this supramolecular gel‐mediated approach for the enhanced intranasal delivery of l‐DOPA with increased bioavailability (higher uptake in the brain). Formulations such as this can potentially be applied to other neurological drugs to treat a wider range of neurodegenerative conditions.

## 3 Conclusions

In summary, we report a simple two‐component supramolecular hydrogel that has appropriate rheological performance and can undergo rapid self‐healing, making it suitable for use in nasal drug delivery. The gel can be loaded with l‐DOPA, our model drug in this study, without disturbing the gel structure. l‐DOPA is largely mobile within the liquid‐like phase of the gel and the mobile l‐DOPA undergoes release from the gel with rapid kinetics. In vitro studies have demonstrated the cytocompatibility of these gels. In vivo studies have indicated that the gel formulation assists with retention in the nasal cavity, limits passage of the drug into the GI tract and subsequent liver metabolism, and enhances levels of blood and brain uptake. Specifically, the nasal delivery gel achieved 4.1 times more brain uptake of l‐DOPA and 2.1 times more l‐DOPA in the blood 10 min after administration than intravenous delivery of an equivalent dose. The gel formulation was also significantly more effective than the administration of a simple l‐DOPA solution intranasally.

We therefore suggest that this gel may have potential in nasal delivery, and that more widely, self‐assembling gels with self‐healing characteristics based on low‐molecular‐weight systems are a suitable low‐cost formulation technology for this application. The ability to rapidly achieve effective levels of drug in the brain, avoiding first pass liver metabolism, suggests that this supramolecular gel‐mediated intranasal drug delivery approach may have a great value in delivering a wide range of therapeutics for treating different difficult‐to‐treat, debilitating, and costly brain disorders such as brain malignancies, neurodegenerative diseases, and psychiatric diseases. Future work would need to focus on measurement and optimization of the system in terms of achieving therapeutic benefit whilst minimizing toxicity, a fuller pharmacokinetic survey across multiple timepoints, development for operation within nasal spray devices and the incorporation of other additives into these simple formulations to potentially further enhance the delivery and half‐life of the active pharmaceutical ingredient.

## 4 Experimental Section

Full details of materials, methods, and additional data can be found in Supporting Information.

### Statistical Methods

For in vitro and in vivo experiments, data are presented as mean ± SD, where *n* denotes the number of repeats. Significant differences were examined using one‐way ANOVA using GraphPad Prism 8. *p* < 0.05 was considered statistically significant in all studies.

### In Vivo Brain and Organ Biodistribution Studies

All animal experiments were performed in compliance with the UK Animals (Scientific Procedures) Act 1986 and UK Home Office Code of Practice for the Housing and Care of Animals Used in Scientific Procedures (Home Office 1989). In vivo experimentation was adhered to the project license approved by the King's College London animal welfare and ethical review body (AWERB) and UK Home Office (PBE6EB195).

## Conflict of Interest

The authors declare no conflict of interest.

## Supporting information

Supporting Information

## Data Availability

Data available in article supplementary material.

## References

[1] a) R. G. Weiss , J. Am. Chem. Soc. 2014, 136, 7519; 24836858 10.1021/ja503363v

[2] a) N. M. Sangeetha , U. Maitra , Chem. Soc. Rev. 2005, 34, 821; 16172672 10.1039/b417081b

[3] a) G. Verma , P. A. Hassan , Phys. Chem. Chem. Phys. 2013, 15, 17016; 23907560 10.1039/c3cp51207j

[4] a) C. Valéry , M. Paternostre , B. Robert , T. Gulik‐Krzywicki , T. Narayanan , J.‐C. Dedieu , G. Keller , M.‐L. Torres , R. Cherif‐Cheikh , P. Calvo , F. Artzner , Proc. Natl. Acad. Sci., U. S. A. 2003, 100, 10258; 12930900 10.1073/pnas.1730609100 PMC193548

[5] a) S. Cao , X. Fu , N. Wang , H. Wang , Y. Yang , Int. J. Pharm. 2008, 357, 95; 18329200 10.1016/j.ijpharm.2008.01.054

[6] a) F. Plourde , A. Motulsky , A. C. Couffin‐Hoarau , D. Hoarau , H. Ong , J. C. Leroux , J. Controlled Release 2005, 108, 433; 10.1016/j.jconrel.2005.08.016 16182402

[7] a) H. Alsaab , S. P. Bonam , D. Bahl , P. Chowdhury , K. Alexander , S. H. S. Boddu , J. Pharm. Pharm. Sci. 2016, 19, 252; 27518174 10.18433/jpps.v19i2.27641

[8] a) L. Illum , J. Controlled Release 2003, 87, 151; 10.1016/s0168-3659(02)00363-2 12618035

[9] a) C. Prego , M. Garcia , D. Torres , M. J. Alonso , J. Controlled Release 2005, 101, 151; 10.1016/j.jconrel.2004.07.030 15588901

[10] a) S. V. Dhuria , L. R. Hanson , W. H. Frey , J. Pharm. Sci. 2010, 99, 1654; 19877171 10.1002/jps.21924

[11] a) Y. Fan , M. Chen , J. Zhang , P. Maincent , X. Xia , W. Wu , Crit. Rev. Ther. Drug Carrier Syst. 2018, 35, 433; 30317945 10.1615/CritRevTherDrugCarrierSyst.2018024697

[12] a) P. A. LeWitt , Mov. Disord. 2015, 30, 64; 25449210 10.1002/mds.26082

[13] F. Amjad , D. Bhatti , T. L. Davis , O. Oguh , R. Pahwa , P. Kukreja , J. Zamudio , L. V. Metman , Adv. Ther. 2019, 36, 2233. 31278691 10.1007/s12325-019-01014-4 PMC6822848

[14] a) S. Charlton , N. S. Jones , S. S. Davis , L. Illum , Eur. J. Pharm. Sci. 2007, 30, 295; 17223022 10.1016/j.ejps.2006.11.018

[15] a) B. A. Aderibigbe , Pharmaceutics 2018, 10, 40; 10.3390/pharmaceutics10020040 PMC6027251 29601486

[16] a) G. Fichman , L. Adler‐Abramovich , S. Manohar , I. Mironi‐Harpaz , T. Guterman , D. Seliktar , P. B. Messersmith , E. Gazit , ACS Nano 2014, 8, 7220; 24936704 10.1021/nn502240r PMC4108209

[17] K. Pathak , Int. J. Pharm. Investig. 2011, 1, 62. 10.4103/2230-973X.82383 PMC3465125 23071923

[18] K. Hawkins , A. K. Patterson , P. A. Clarke , D. K. Smith , J. Am. Chem. Soc. 2020, 142, 4379. 32023044 10.1021/jacs.9b13156 PMC7146862

[19] A. Andersen , Int. J. Toxicol. 2006, 25, 11.16835129

[20] Frequency of Use of Cosmetic Ingredients, Food and Drug Administration, Washington, D.C. 2001.

[21] S. Laham , B. Broxup , M. Robinet , M. Potvin , K. Schrader , Am. Ind. Hyg. Assoc. J. 1991, 52, 503. 1781429 10.1080/15298669191365126

[22] a) B. Escuder , M. Llusar , J. F. Miravet , J. Org. Chem. 2006, 71, 7747; 16995682 10.1021/jo0612731

[23] C.‐V. Nguyen , F. Li , H. Li , B. S. Wong , C. Y. Low , X.‐Y. Liu , L. Kang , Mol. Pharm. 2015, 12, 44. 10.1021/mp500542a 25495699

[24] a) X. Yu , L. Chen , M. Zhang , T. Yi , Chem. Soc. Rev. 2014, 43, 5346; 24770929 10.1039/c4cs00066h

[25] a) G. M. Eccleston , M. Bakhshaee , N. E. Hudson , D. H. Richards , Drug Dev. Ind. Pharm. 2000, 26, 975; 10914322 10.1081/ddc-100101325

[26] E. V. Brovč , J. Mravljak , R. Šink , S. Pajk , Int. J. Pharm. 2020, 581, 119285. 32240804 10.1016/j.ijpharm.2020.119285

[27] a) C. Gloxhuber , Arch. Toxicol. 1974, 32, 245; 4614759 10.1007/BF00330108

[28] a) T. K. Kim , W. Kang , I. K. Chun , S. Y. Oh , Y. H. Lee , H. S. Gwak , Eur. J. Pharm. Sci. 2009, 8, 525; 10.1016/j.ejps.2009.09.019 19804823

[29] Y. C. Wong , Z. Zuo , Pharm. Res. 2010, 27, 1208. 20372990 10.1007/s11095-010-0127-5

[30] H. Shindo , N. Miyakoshi , I. Takahashi , Chem. Pharm. Bull. 1971, 19, 2490. 10.1248/cpb.19.2490 5145918

[31] P. Y. Gambaryan , I. G. Kondrasheva , E. S. Severin , A. A. Guseva , A. A. Kamensky , Exp. Neurobiol. 2014, 23, 246. 25258572 10.5607/en.2014.23.3.246 PMC4174616

[32] a) M. Dahlin , U. Bergman , B. Jansson , E. Björk , E. Brittebo , Pharm. Res. 2000, 17, 737; 10955850 10.1023/a:1007542618378

[33] M. A. de Souza Silva , C. Mattern , R. Häcker , P. J. C. Nogueira , J. P. Huston , R. K. W. Schwarting , J. Neurochem. 1997, 68, 233. 8978730 10.1046/j.1471-4159.1997.68010233.x

[34] M. A. de Souza Silva , C. Mattern , R. Häcker , C. Tomaz , J. P. Huston , R. K. W. Schwarting , Synapse 1997, 27, 294. 9372552 10.1002/(SICI)1098-2396(199712)27:4<294::AID-SYN3>3.0.CO;2-7

[35] a) S. Shrewsbury , J. Campbell , M. Swardstrom , A. Lehn , K. Satterly , J. Hoekman , Mov. Disord. 2019, 34, Suppl 2;

