# Supplementary material for: Enhanced Delivery of Neuroactive Drugs via Nasal Delivery with a Self‐Healing Supramolecular Gel
Source: Adv Sci (Weinh). 2021 May 24;8(14):2101058. doi: 10.1002/advs.202101058 (PMC8292877; doi:10.1002/advs.202101058)
Supplement: Supplementary file 1 — Supporting Information [file ADVS-8-2101058-s001.pdf]

© 2021 Wiley-VCH GmbH

ADVANCED SCIENCE
Open Access

Supporting Information

for *Adv. Sci.*, DOI: 10.1002/advs.202101058

Enhanced Delivery of Neuroactive Drugs via Nasal Delivery with a Self-Healing Supramolecular Gel

Julie Tzu-Wen Wang, Ana C. Rodrigo, Anna K. Patterson, Kirsten Hawkins, Mazen M. S. Aly, Jia Sun, Khuloud T. Al Jamal and David K. Smith**

Supplementary information

Enhanced Delivery of Neuroactive Drugs via Nasal Delivery with a Self-Healing Supramolecular Gel

Julie Tzu-Wen Wang[a], Ana C. Rodrigo[b], Anna K. Patterson[b], Kirsten Hawkins[b], Mazen M. S. Aly[a], Jia Sun[a], Khuloud T. Al Jamal[a,] and David K. Smith[b,]**

(a) Dr. J. T. Wang, Mr. M. M. S. Aly, Dr. J. Sun, Prof K. T. Al-Jamal*
Institute for Pharmaceutical Science, King's College London, London SE1 9NH, UK
Email: khuloud.al-jamal@kcl.ac.uk

(b) Dr. A. C. Rodrigo, Ms. A. K. Patterson, Dr K. Hawkins, Prof D. K. Smith*
Department of Chemistry, University of York, Heslington, York, YO10 5DD, UK
E-mail: david.smith@york.ac.uk

Contents

1. Materials and General Methods
2. Formation of Hydrogels to Test Maximum Loading of L-DOPA
3. Imaging of Hydrogels with L-DOPA
4. NMR studies of Hydrogels
5. Rheology of Hydrogels
6. Hydrogels and Buffers
7. Additional Biological Data
8. References

1. Materials and General Methods

All compounds required for synthesis and analysis were purchased from standard chemical suppliers and used without further purification. The glutamine amide was synthesised according to the method previously disclosed by us.[1] For biological studies, Eagle's Minimum Essential Medium (MEM), fetal bovine serum (FBS), penicillin/streptomycin, Trypsin/EDTA, glutaMAX, and phosphate buffered saline (PBS) were obtained from Gibco, Thermo Fisher Scientific Inc. (UK). MTT Formazan powder and CTAB were purchased from Sigma-Aldrich (UK). L-3,4-dihydroxyphenyl alanine (L-DOPA), 98+% was obtained from Alfa Aesar, UK. Tween®80 and DMSO were obtained from Fisher Scientific. [^{3}H] L-3,4-dihydroxyphenyl alanine, [phenyl-2,5,6-3H] 250µCi @ 500µCi/mL in 0.2N HOAc/EtOH with specific radioactivity activity > 44Ci/mmol was purchased from ViTrax, USA. Isoflurane (IsoFlo®) for anesthesia was purchased from Abbott Laboratories Ltd (UK). Phenobarbital (200 mg in 1 mL solution for injection Phenobarbital Sodium) was obtained from Boehringer Ingelheim Animal Health, UK. Soluene®-350 tissue solubiliser was purchased from PerkinElmer. Scintisafe scintillation cocktail (formerly known as Optiphase safe), sodium chloride, isopropanol and hydrogen peroxide (30 % w/v) were obtained from Fisher Scientific. Acetic acid (glacial) 100 % was obtained from Merck. ^{1}H NMR were recorded on a Jeol 400 spectrometer (^{1}H 400 MHz). Coupling constants (*J*) are recorded in Hz. All rheological measurements were carried out using a Malvern Instruments Kinexus Pro+ rheometer. T_{gel} values were recorded using a high precision thermoregulated oil bath. UV-vis absorbance was measured on a Shimadzu UV-2401 PC spectrophotometer.

2. Formation of Hydrogels to Test Maximum Loading of L-DOPA

Glutamine amide (3.5 mg) and benzaldehyde (1.13 µl) were added to a sample vial, along with a known mass of L-DOPA (Table S1). Deionised water (1 ml) was added, and the resulting mixture heated until all the solid was dissolved. The solution was then left to cool, and the formation of a gel checked using the tube inversion test.

Table S1. Effect of L-DOPA loading on the gel formed by two-component gelator **1**. TG=Transparent Gel, G (I)=not all solid dissolved, but gel still formed.

L-DOPA / mg	Ratio (Benzaldehyde:L-DOPA)	Outcome
0.55	1:0.25	TG
1.07	1:0.5	TG
1.65	1:0.75	TG
2.19	1:1	TG
4.42	1:2	TG
6.63	1:3	TG
8.81	1:4	TG
11.73	1:5	G (I)
23.74	1:10	G (I)

3. NMR studies of Hydrogels

All NMR studies were carried out in triplicate.

Determination of unincorporated benzaldehyde: Glutamine amide (3.5 mg) and benzaldehyde (1.13 µl) were added to a sample vial, and D_2O (1 ml) added, along with DMSO (2 µl) as an internal standard. This was then heated until the solids were dissolved, and transferred rapidly to a warm NMR tube. This was then allowed to cool, and a gel formed.

Determination of unbound L-DOPA: Glutamine amide (3.5 mg) and benzaldehyde (1.13 µl) were added to a sample vial, along with a known mass of L-DOPA. D_2O (1 ml) was then added, along with DMSO (2 µl) as an internal standard. The mixture was heated until the solids were dissolved, and transferred rapidly to a warm NMR tube. This was then allowed to cool, and a gel formed.

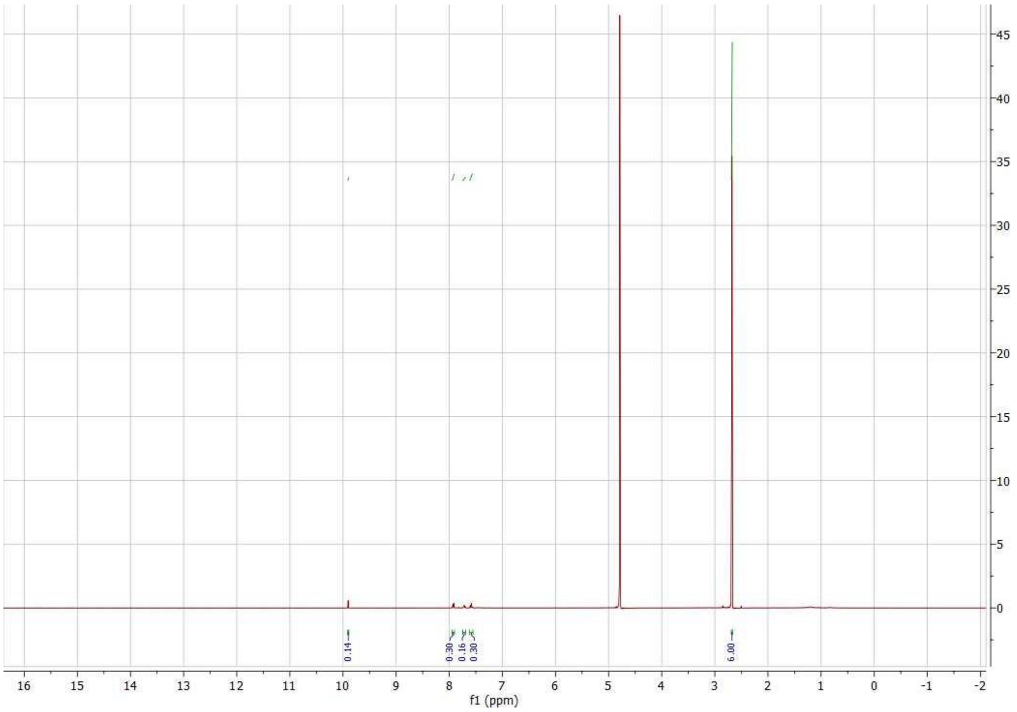

Figure S1a. Sample ^{1}H NMR spectrum for determination of unincorporated benzaldehyde (CHO peak, ca. 10ppm, aromatic peaks 7.0-8.0 ppm, DMSO peak ca. 2.5 ppm).

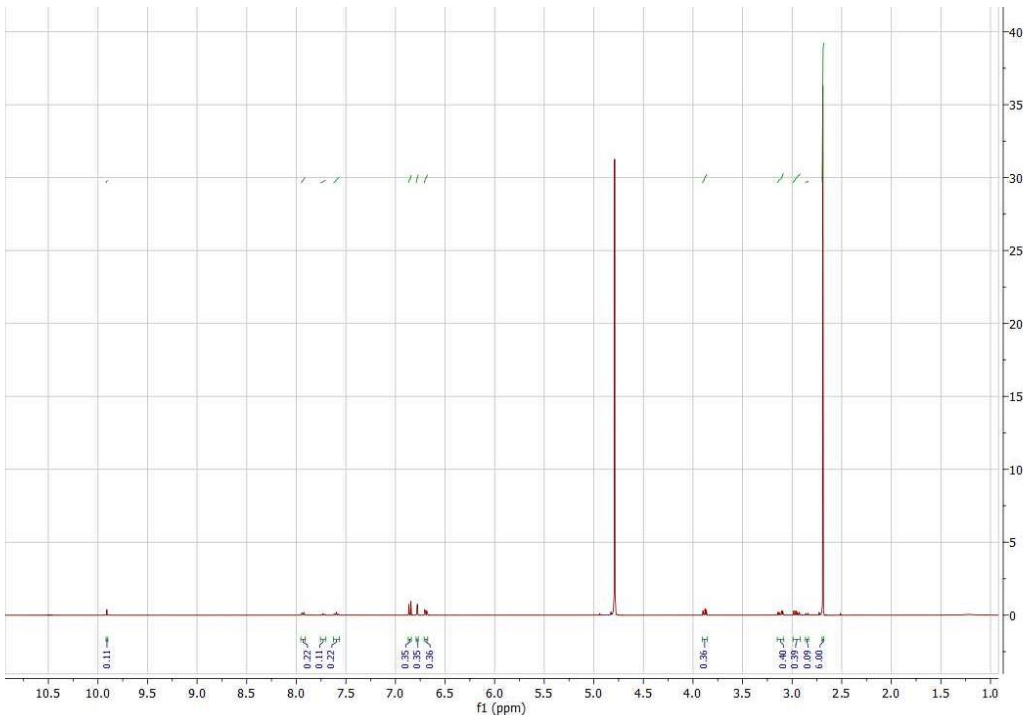

Figure S1b. Sample ^{1}H NMR Spectrum for determining percentage of ‘free’ L-DOPA (aromatic peaks 6.5-7.0 ppm, aliphatic peaks 4.0-3.0 ppm, DMSO peak ca. 2.5 ppm).

4. Rheology of Hydrogels

Formation of hydrogels for rheology: Glutamine amide (3.5 mg) and benzaldehyde (1.13 µl) were added to a sample vial. For hydrogels with L-DOPA, a known mass of L-DOPA was also added at this stage. Deionised water (1 ml) was then added, and the mixture heated until the solid had dissolved. The solution was then quickly transferred to a warm bottomless vial attached to a plate. The solution was allowed to cool down, and the resulting gel disc could be transferred to the rheometer for analysis. All rheology measurements were carried out in triplicate. For the recovery experiment, initially a shear force of 0.0126% was applied, with a frequency of 2 Hz, for 200 seconds. The frequency was then increased to 100 Hz for 30 seconds. The frequency was then returned to 2 Hz.

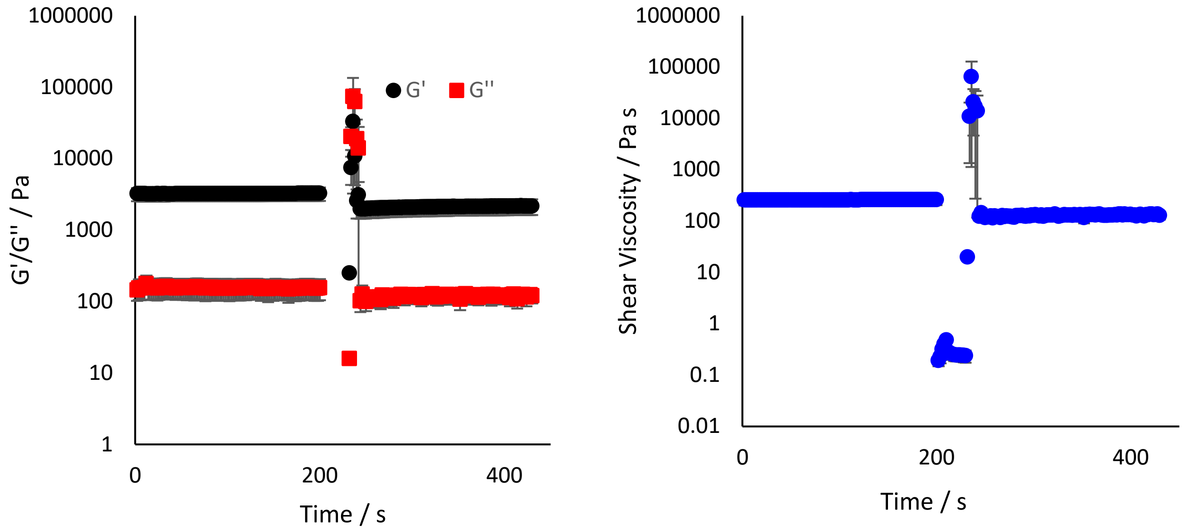

Figure S2. Viscoelasticity (left) and viscosity (right) for glutamine amide gels (0.46% wt/vol) without L-DOPA (1:1 molar ratio with benzaldehyde).

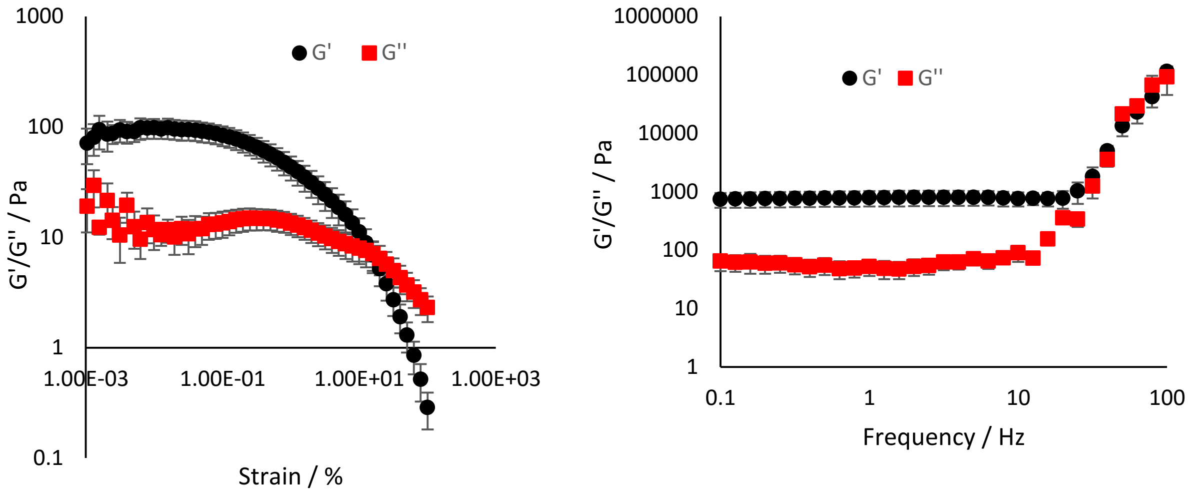

Figure S3. Amplitude sweep (left) and frequency sweep (right) for hydrogels (0.46% wt/vol) with L-DOPA (1:1 molar ratio with benzaldehyde).

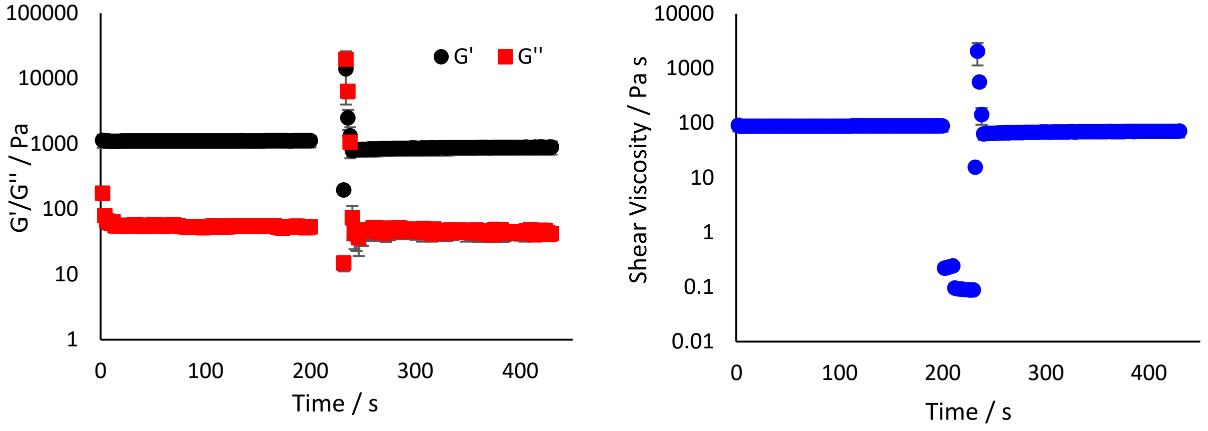

Figure S4. Viscoelasticity (left) and viscosity (right) for glutamine amide gels (0.46% wt/vol) with L-DOPA (1:1 molar ratio with benzaldehyde).

5. Imaging of Hydrogels with L-DOPA

Imaging was carried out by Meg Stark, at the Biology Technology Facility, Department of Biology, University of York. TEM samples were prepared using the following method: A small portion of gel was transferred, by drop-casting, to a heat-treated copper support. Excess material was removed using a filter paper, and the samples air-dried for 20 minutes. TEM images were recorded on a FEI Technai 12 G2. SEM samples were prepared using the following method: A small portion of gel was transferred to a copper support, then freeze-dried by plunging into liquid nitrogen. The samples were then lyophilised for 12 hours, and any excess material removed. The dried sample was then sputter coated with a thin layer of gold/palladium, to prevent sample charging, and imaging carried out. SEM images were recorded on a JEOL JSM-6490LV.

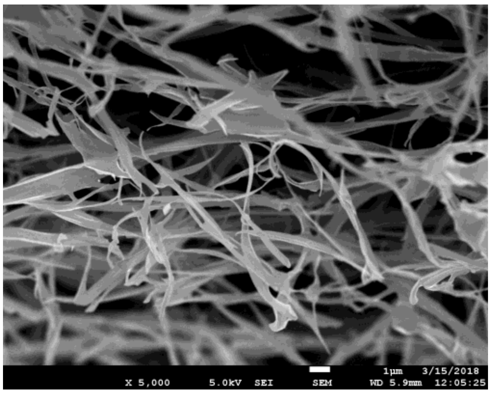

Figure S5. SEM image for a glutamine amide hydrogel, Scale bar 1 μm.

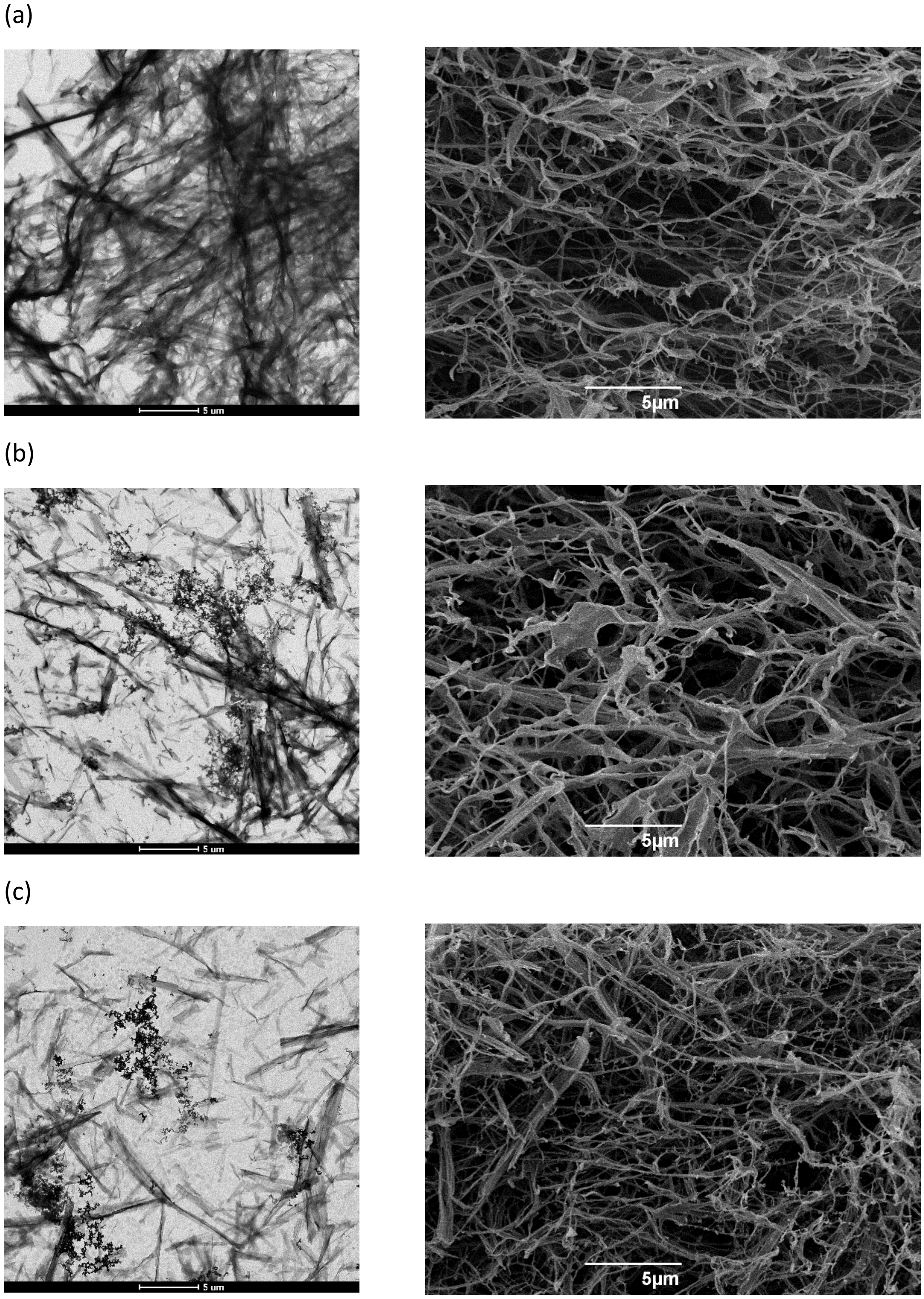

Figure S6. TEM (left) and SEM (right) images for glutamine amide gels with L-DOPA. (a) benzaldehyde:L-DOPA 1:1 (b) benzaldehyde:L-DOPA 1:4 (c) benzaldehyde:L-DOPA 1:10. Dark aggregates (which we assign as L-DOPA) can be observed in the TEM images at higher loadings of L-DOPA (b and c). All scale bars 5 μm.

6. Stability of hydrogels in relevant conditions.

Testing formation of hydrogels in other relevant solutions: Glutamine amide (3.5 mg) and benzaldehyde (1.13 µl) were added to a sample vial, and the relevant solution (1 ml) added (see Table S for details). The mixture was then heated until the solid was dissolved. The solution was left to cool, and the formation of a gel determined by the tube inversion test.

Table S2. Testing formation of hydrogels in PBS and glucose solution. P=Precipitate, G=Gel.

Solution	Outcome
pH 7.4 PBS (0.01 M)	P
PBS without Ca/Mg (1X)	P
Glucose (5% solution)	G

Testing stability of gels to PBS and glucose solution: The hydrogels were prepared, with deionised water, according to the standard method. Once the gels had formed, the relevant solution (1 ml) was added (see Table S for details). The gels were then monitored to determine their stability to the solution.

Table S3. Monitoring the stability of the glutamine amide hydrogels to relevant solutions.

Solution	Outcome
pH 7.4 PBS (0.01 M)	Remained a gel for 48+ hours
PBS without Ca/Mg (1X)	Remained a gel for 48+ hours
Glucose (5% solution)	Remained a gel for 48+ hours

7. Biological Studies and Additional Biological Data

***In vitro* cytotoxicity studies.** RPMI 2650 human nasal septum tumour cells (ATCC® CCL-30™), were cultured in MEM medium supplemented with 10% FBS, 50 U/mL penicillin, 50 µg/mL streptomycin and 1% L-GlutaMAX. Cells were incubated in 5% CO_2 at 37 °C. Cells were routinely grown in 75 cm^2 canted-neck tissue culture flasks and passaged using Trypsin/EDTA at 80 % confluence. To assess the cytotoxicity of the L-DOPA loaded hydrogel, RPMI 2650 cells were seeded in 96-well plates at the density of 40,000 cells/well over night. A blank gel (3.5 mg/mL) and a gel loaded with 4.8 mM L-DOPA were formulated to settled overnight. Solutions of L-DOPA (4.8 mM), CTAB (3.5 mg/mL) and Tween®80 (3.5 mg/mL) were also prepared. Cells were incubated with culture medium containing 1, 2, 5 and 10 % (v/v) of the two gels, L-DOPA, CTAB or Tween®80 solutions for 24 and 48 h. Cytotoxicity was examined by the standard MTT assay. In brief, at the end of the incubation period, the media was removed and replaced with MTT solution prepared in culture medium (0.5 mg/mL). Cells were incubated for 3 h at 37 °C and 5 % CO_2. The formed formazan was dissolved in DMSO (200 µL) and the plate was read at 570 nm in a FLUO star OPTIMA plate reader (BMG Labtech). The results were calculated by normalization to untreated cells and are expressed as the percentage relative viability (%) as mean ± SD.

***In vivo* brain and organ biodistribution studies.** All animal experiments were performed in compliance with the UK Animals (Scientific Procedures) Act 1986 and UK Home Office Code of Practice for the Housing and Care of Animals Used in Scientific Procedures (Home Office 1989). *In vivo* experimentation was adhered to the project licence approved by the King's College London animal welfare and ethical review body (AWERB) and UK Home Office (PBE6EB195). [^{3}H]L-DOPA hydrogel/solution for intranasal administration was formulated as 4.8 mM and 75 µCi/mL. Female Balb/c mice were anaesthetised by isoflurane inhalation and intranasally administered with [^{3}H]L-DOPA hydrogel/solution by injecting 2 µL per administration to the left and right nostrils alternatively at a 20 s interval for a total of 20 µL (L-DOPA 0.95 mg/kg, 1.5 µCi/mouse) to allow sufficient retention of gel in nose and prevent dripping. At 10 min, 20 min and1 h post administration (n=3), mice were given overdosed phenobarbital and venous blood was collected from vena cava. Whole body perfusion was

then performed using 0.9 % saline through the heart to wash out the blood. Selected major organs (brain, heart, lung, liver, spleen, and kidneys) and nasal cavity were harvested and weighted. For intravenous injection, mice (n=3) were anaesthetised by isoflurane inhalation and intravenously administered with 100 µL of [^{3}H]L-DOPA saline solution (0.95 mg/kg, 1.5 µCi/mouse) via a tail vein. At 10 min post injection, mice were humanely killed and same tissue samples were collected by the same methods described above. All tissues including blood were solubilised with Soluene®-350 in scintillation vials, followed by heating at 55 °C overnight. Solubilised tissues were bleached with H_2O_2 and isopropanol, followed by heating at 55 °C for at least 3 h. Scintillation cocktail (10 mL) was added and the vials were stored in dark for 24 h followed by liquid scintillation counting (LS 6500 Multi-Purpose Scintillation Counter, Beckman Coulter). Control tissues from naïve mice spiked with known dose of [^{3}H]L-DOPA were proceeded with the same steps for scintillation counting for measurement normalization.

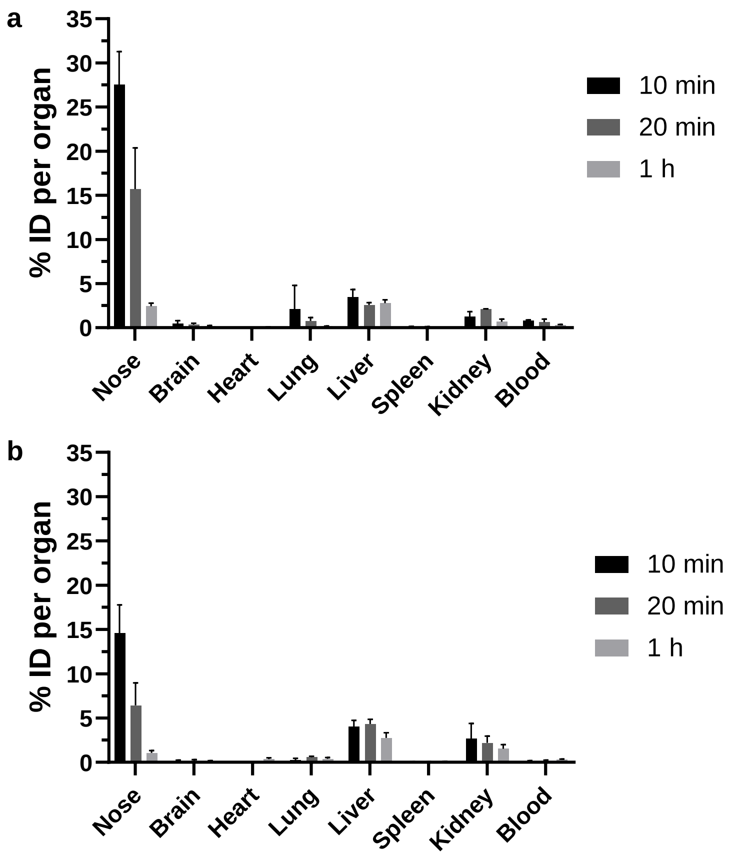

Figure S7. Organ biodistribution of [^{3}H]L-DOPA formulated in hydrogel or solution in mice at 10 min, 20 min and 1 h after intranasal administration. Results expressed as mean ± SD, n=3.

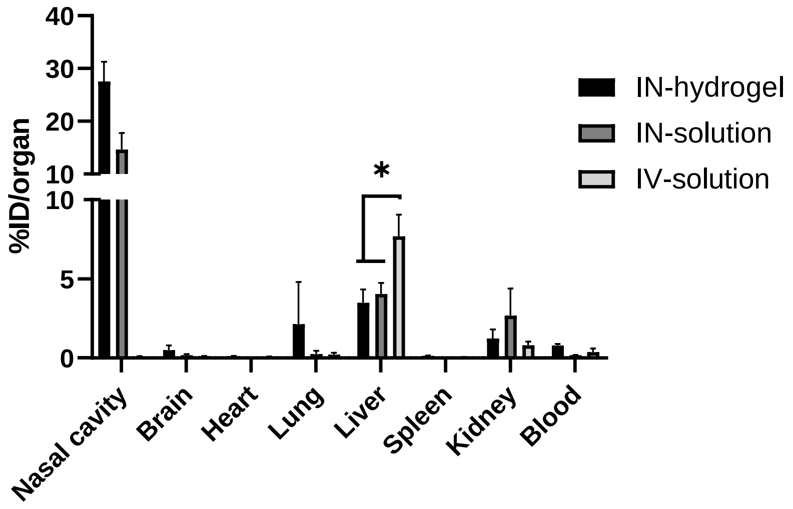

Figure S8. Organ biodistribution of [^{3}H]L-DOPA formulated in hydrogel or in solution in mice at 10 min after intranasal (IN) or intravenous (IV) administration. Results are expressed as mean ± SD, n=3. *p < 0.05 (t-test).

Brain distribution studies. Three mice received [^{3}H]L-DOPA hydrogel (L-DOPA 0.95 mg/kg, 1.5 µCi/mouse) nasally. At 10 min post injection, the brains were removed following transcardial perfusion with 0.9 % saline under terminal anaesthesia. The different brain segments (the olfactory bulbs (OB), cerebrum (CB), brain stem (BS), cerebellum (CE), spinal cord (SP) and trigeminal nerves (TN)) were dissected and proceeded with liquid scintillation counting as described previously.

Statistical Methods. For *in vitro* and *in vivo* experiments, data are presented as mean ± SD, where *n* denotes the number of repeats. Significant differences were examined using one-way ANOVA using GraphPad Prism 8. p<0.05 was considered statistically significant in all studies.

8. References

1. K. Hawkins, A. K. Patterson, P. A. Clarke, D. K. Smith, *J. Am. Chem. Soc.*, **2020**, *142*, 4379-4389.
